# seneR: An R package for comprehensive senescence assessment and its application in type 2 diabetes and osteoarthritis

**DOI:** 10.1016/j.csbj.2025.12.031

**Published:** 2025-12-31

**Authors:** Yi Zhang, Xinming Zhang, Cheng Chen, Bing Li, Yao Lu, Xin Ma, Yunfeng Yang

**Affiliations:** a Department of Orthopedics, Tongji Hospital, School of Medicine, Tongji University, Shanghai 200092, China; b Department of Chemistry, Rice University, Houston, TX 77005, USA; c Department of Orthopaedics, Shanghai Sixth People’s Hospital Affiliated to Shanghai Jiao Tong University, School of Medicine, 600 Yishan Rd, Shanghai 200233, China; d Department of Orthopedics, Ruijin Hospital, Shanghai Jiao Tong University School of Medicine, Shanghai, China; e Department of Microbiology and Immunology, Mcgill University, Canada

**Keywords:** Cellular senescence, Transcriptome analysis, Type 2 Diabetes, Osteoarthritis, Phentolamine

## Abstract

**Background:**

Cellular senescence is a key driver of aging and chronic diseases. However, accurately identifying senescent cells is challenging due to limitations of conventional biomarkers and senescence heterogeneity. Transcriptome-wide analyses offer powerful tools for deciphering cellular states. Yet, there is a critical gap in computational frameworks for senescence assessment from transcriptomic data.

**Methods:**

We developed the seneR package, which includes functions such as calculating senescence identity scores (SID scores), assessing senescence-related phenotypes, and plotting senescence trajectories, and provides an interactive Shiny interface. We applied seneR to transcriptome datasets from human islets and chondrocytes to investigate the role of senescence in Type 2 Diabetes (T2D) and osteoarthritis (OA). Additionally, in vitro validation confirmed phentolamine (PM)'s potential to delay chondrocyte senescence.

**Results:**

seneR accurately identified senescent cells and revealed senescence-related phenotypes in transcriptome datasets. In T2D, SID scores were significantly higher in elderly islets. Senescent islet cells exhibited diminished responsiveness to nutrient stimuli, linking senescence to impaired insulin secretion. In OA, seneR identified SLPI as a molecule strongly associated with chondrocyte senescence, with PM treatment reducing SID scores. Trajectory analysis revealed chondrocyte senescence progression and potential therapeutic targets. In vitro experiments, PM reversed both IL-1β- and H₂O₂-induced chondrocyte senescence.

**Conclusion:**

Our study demonstrates that seneR is a valuable tool for assessing cellular senescence from transcriptomic data, revealing key phenotypes and potential therapeutic targets in T2D and OA. The identification of SLPI as a senescence-associated molecule and the therapeutic potential of PM highlights the utility of our approach in understanding senescence-related diseases.

## Graphical Abstract

**Figure:**
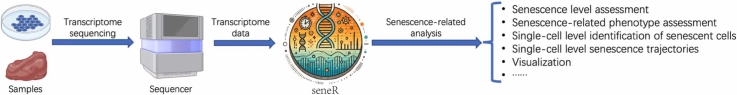

## 1 introduction

Cellular senescence (CS) is a state of stable and essentially irreversible cell-cycle arrest triggered by a range of stressors, including DNA damage, oncogenic activation, and oxidative stress [1], [2], [3], [4]. The concept of cellular senescence was initially elucidated by Hayflick through studies on human fibroblasts undergoing repeated subculturing [5]. Over the past decades, the accumulation of senescent cells has been increasingly recognized as a key driver of chronic inflammation, tissue dysfunction, and the pathogenesis of several age-related diseases [6], [7]. For example, recent studies indicate that cellular senescence of pancreatic β cells impairs insulin secretion, while specific deletion of senescent cells (senolysis) improves β cell function and blood glucose levels [8], [9]. Similarly, in osteoarthritis (OA), senescent chondrocytes promote cartilage degradation by secreting pro-inflammatory cytokines and matrix-degrading enzymes (collectively known as the senescence-associated secretory phenotype (SASP)).

Despite its physiological significance, the accurate identification of senescent cells remains challenging due to limitations of conventional biomarkers: protein markers (e.g., p16^INK4a^, p21^WAF−1/CAP1^) exhibit context-dependent expression, while histochemical assays such as SA-β-gal staining suffer from subjective interpretation and technical variability [10], [11], [12]. These issues are further complicated by the inherent heterogeneity of senescence, which varies depending on cell type, tissue, and the inducing stressor [13]. Therefore, a more comprehensive approach, such as transcriptome-wide analysis, is needed to better understand and identify senescent cells.

Transcriptome-wide analyses have emerged as powerful tools for deciphering cellular states. In particular, the advent of single-cell RNA sequencing (scRNA-seq) has revolutionized the study of cellular heterogeneity. By enabling transcriptomic profiling at single-cell level, it provides unprecedented insights into complex cellular states, including senescence. However, a critical gap remains in computational frameworks designed explicitly for senescence assessment from transcriptomic data.

Recently, several Python-based tools, such as SenCID and SenePy, have leveraged machine learning to identify senescent cells based on transcriptomic signatures [13], [14]. These models have demonstrated utility in studying senescence across diverse biological contexts, from normal aging to chronic diseases. However, their limited compatibility with R, a widely used language in the bioinformatics community, hinders their integration into existing R-based workflows, such as Seurat and SingleCellExperiment [15], [16]. Additionally, many existing Python-based tools rely on strict dependencies tied to specific versions of Python and associated libraries, which are often incompatible with common operating systems used by researchers, such as macOS and Windows. These constraints limit researchers’ ability to seamlessly incorporate senescence assessments into their bioinformatics workflows, especially for users with limited experience in Python.

To address this gap, we developed seneR, an R package that integrates the SenCID model, enhancing its accessibility and functionality within the R ecosystem. This adaptation not only improves compatibility with popular R packages but also introduces several new functionalities, including the assessment of senescence-related phenotypes and the plotting of senescence trajectories. To demonstrate the utility of seneR, we conducted two case studies. In the Type 2 Diabetes (T2D) case study, we demonstrated how senescence in pancreatic islets influences insulin secretion patterns. In the OA case study, we identified the SLPI gene and phentolamine (PM) as significant modulators of chondrocyte senescence, which we further validated in vitro. These findings not only highlight the potential of seneR in senescence-related research but also provide new therapeutic targets for OA.

## 2 Results

### 2.1 seneR package

**Fig. 1:**
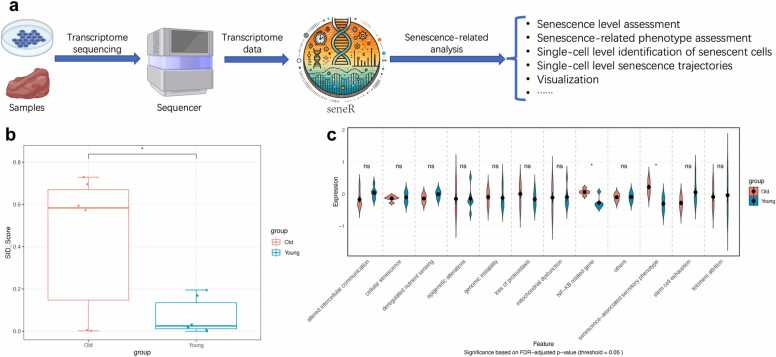
Schematic diagram and example analysis of seneR. (a) Schematic diagram of the workflow for analysis using seneR. (b) Boxplot showing the difference in SID scores between the young group and the old group. (c) Violin plot showing differences in multiple senescence-related phenotypes between young and old groups. Data are presented as mean ± standard deviation (n = 6). *p < 0.05; **p < 0.01; ***p < 0.001; ****p < 0.0001; ns, not significant. (*t*-test: b; *t*-test with FDR correction: c).

We developed the seneR package (https://github.com/dr-yi-zhang/seneR) to conduct senescence-related analyses of transcriptomic data (including bulk and single cell RNA-seq). It incorporates the SenCID predictive model described initially by Tao et al. [14], whose efficacy was validated extensively in their study (distributed under an MIT License). In essence, the SID score model can select appropriate senescence patterns for different cell types and assign corresponding senescence scores (senescence identity score, SID score). In addition, the seneR package provides a comprehensive suite of functions for senescence-related analyses. These include evaluating multiple senescence-associated phenotypes, data visualization, and analysis of single-cell transcriptomic datasets (Fig. 1a). In addition to the R functions, we provide a user-friendly Shiny application (https://lzy-rproject.shinyapps.io/seneR-analysis-portal/) that enables researchers and clinicians to reproduce all analyses without requiring any programming skills. Furthermore, the seneR package includes detailed vignettes (https://github.com/dr-yi-zhang/seneR) that guide users through step-by-step analyses. These features enable users to easily and rapidly gain insights into the senescence status of their samples.

To ensure the fidelity of our implementation, we conducted rigorous benchmarking against the original SenCID model. The SID scores generated by seneR showed near-perfect correlation with those from SenCID (R^2^ = 1, Figure S1a), and classification performance metrics were virtually identical (Figure S1a-d).

For example, we applied the seneR package to a bulk RNA-seq dataset of chondrocytes (GSE246425), including young cells (passages 1–3) and senescent cells (serially passaged to 15–18 passages). The SID score showed higher values in the senescent group compared to the young group (Fig. 1b). Assessment of senescence-associated phenotypes revealed that chondrocyte senescence was characterized primarily by activation of the NF-κB pathway and upregulation of the SASP (Fig. 1c). These findings are consistent with previous studies and indicate the reliability of the tool [7], [17], [18]. To further validate seneR, we performed additional benchmarking in the chondrocyte senescence dataset (GSE246425). SID scores showed positive associations with established senescence markers, including CDKN2A, CDKN1A, and SASP factors CCL2 and CCL5 (Figure S1e). Classification performance analysis demonstrated that SID scores can distinguish senescent from young chondrocytes with high precision (AUROC = 0.722; precision = 1.00) (Figure S1e-f), confirming the utility of seneR for reliable senescence assessment.

### 2.2 Case study of Type 2 diabetes (T2D)

**Fig. 2:**
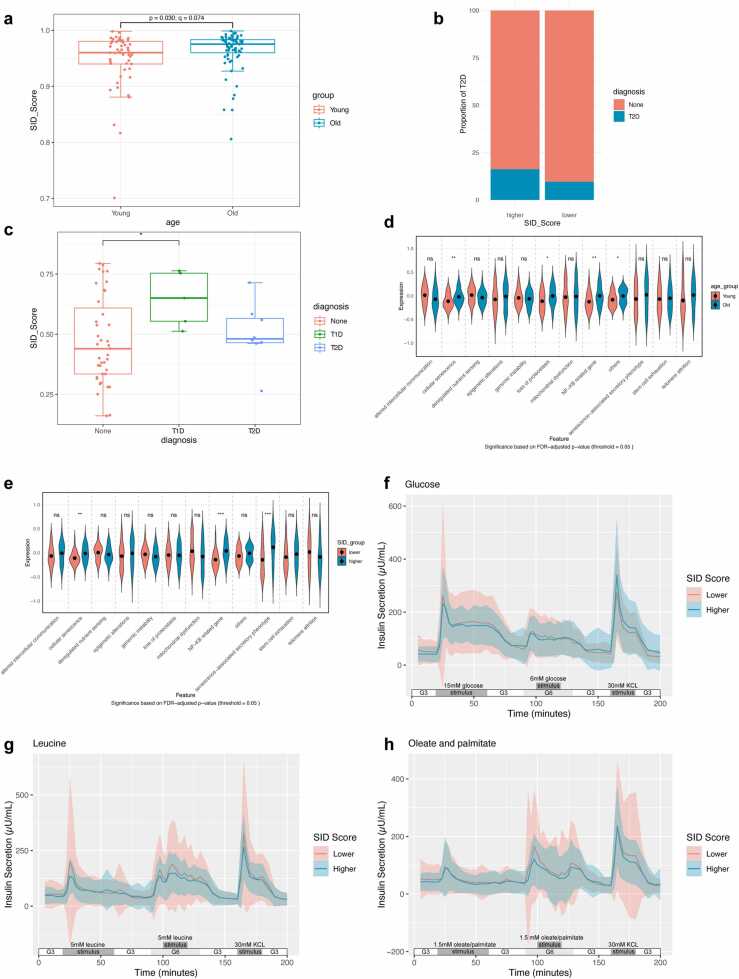
Association of islet senescence with T2D and insulin secretion dynamics. (a) Box plot showing the difference in SID scores between young (≤ median age) and aged (> median age) groups. (Linear mixed-effects model; p: original p value; q: FDR adjusted p value). (b) Proportion of T2D cases in higher vs. lower SID groups. (c) Box plot showing the difference in SID scores among different groups for α cell. (Linear mixed-effects model). (d, e) Violin plot showing differences in multiple senescence-related phenotypes between different groups. (*t*-test with FDR correction). (f-h) Averaged traces of dynamic insulin secretion measurements in response to glucose (15 or 6 mM, f), leucine (5 mM, g), oleate and palmitate (1.5 mM. 1:1 mixture, h) or KCl (30 mM) in islets isolated from donors. The bar above the horizontal axis represents the following time intervals: 3 mM glucose (0–20 min); stimulus (20–60 min); 3 mM glucose (60–90 min); 6 mM glucose (90–130 min); stimulus (100–120 min); 3 mM glucose (130–160 min); 30 mM KCl (160–180 min); 3 mM glucose (180–200 min). The solid line represents the mean, and the shaded part represents mean ± SD. Data are presented as mean ± standard deviation. *p < 0.05; **p < 0.01; ***p < 0.001; ****p < 0.0001; ns, not significant.

Recent studies have shown a significant association between cellular senescence and T2D. Using the tools we developed, we analyzed the islet transcriptomic dataset available from HumanIslets (https://www.humanislets.com/). We employed a linear mixed-effects model to explore the effect of donor characteristics on SID score, with batch as a random effect and age, sex, BMI, and T2D status as fixed effects. After FDR correction, the association between the old age group and higher SID scores showed a trend toward significance (β = 0.0183, 95 % CI [0.0022, 0.0344], q = 0.074; original p = 0.030) (Fig. 2a). The confidence interval excluding zero provides additional support for a positive association consistent with the expected increase in cellular senescence with aging. Neither sex (β = 0.0017, 95 % CI [-0.0146, 0.0180], q = 0.843), BMI group (β = 0.0021, 95 % CI [-0.0136, 0.0177], q = 0.843), nor T2D status (β = −0.0034, 95 % CI [-0.0208, 0.0139], q = 0.843) were significant predictors in this comprehensive model. This finding is consistent with previous studies reporting an age-dependent increase in the proportion of SA-β-gal+ cells in human cadaveric islets [19]. When samples were divided into high and low SID score groups, a higher proportion of T2D cases was observed in the high SID score group compared to the low SID score group (Fig. 2b). Interestingly, when analyzing α cells using pseudobulk RNA-seq, it was found that the SID in the T1D group was significantly higher compared to the non-diabetic group (Fig. 2c).

Assessment of senescence-related phenotypes showed that old islets exhibited significant differences compared to young islets, including proteostasis loss and NF-κB activation (Fig. 2d). High SID score samples shared similarities with the old samples but were not identical, indicating that organismal aging does not equate to cellular senescence at the molecular level (Fig. 2e, S1h).

To explore the impact of senescence on insulin secretion patterns, we analyzed data on the dynamic response of islets to three prototypical nutrients (glucose, leucine, and oleate/palmitate representing carbohydrates, amino acids, and fatty acids, respectively). As shown in Fig. 2f-h, the solid lines (representing the mean value) of the higher SID group mainly lie above or coincide with those of the lower SID group, except for the stimulation of KCL after glucose stimulation (The negative values in the graph are due to subtracting the baseline). Additionally, statistical analysis (Figure S1i) revealed that the Time to peak insulin (min), which can serve as an indicator of islet responsiveness to nutrients, was significantly higher in the higher SID group (25.80 ± 4.06) than in the lower SID group (22.83 ± 7.31) for oleate/palmitate. For glucose and leucine, although no significant differences were observed, the Time to peak insulin (min) values were higher in the higher SID group (glucose: 26.06 ± 1.46 vs. 25.47 ± 4.24; leucine: 25.91 ± 2.75 vs. 25.64 ± 3.92), consistent with the trend observed for oleate/palmitate. These findings suggest that β-cell senescence is associated with a diminished responsiveness to these three nutrient stimuli.

### 2.3 Case study of osteoarthritis (OA)

**Fig. 3:**
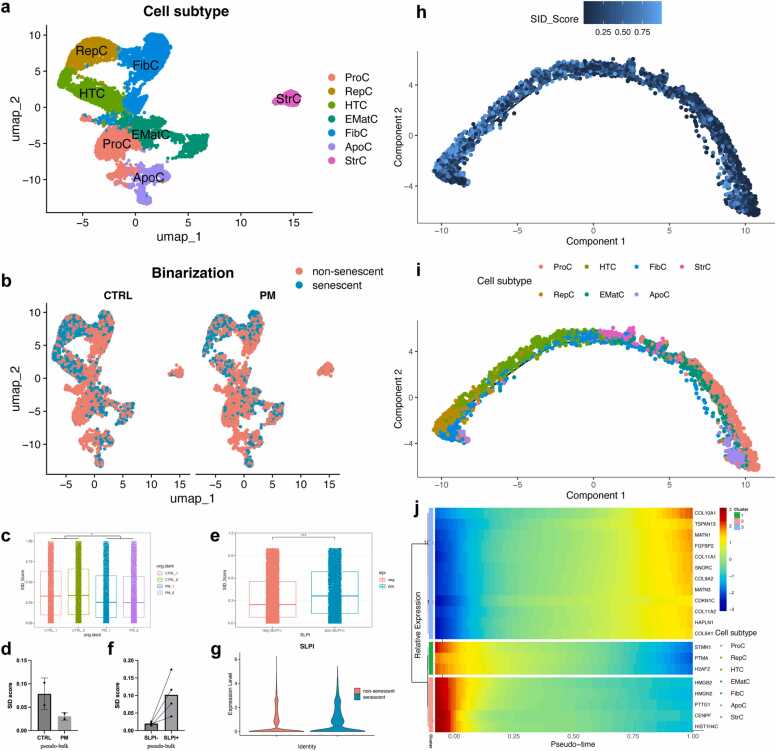
Senescence of chondrocytes was analyzed at the single-cell level using seneR. (a) The visualization of chondrocyte populations using uniform manifold approximation and projection (UMAP). There was a total of seven populations: reparative chondrocytes (RepC), apoptotic chondrocytes (ApoC), hypertrophic chondrocytes (HTC), early mature chondrocytes (EMatC), fibrocartilage chondrocytes (FibC), stressed chondrocytes (StrC), and proliferation chondrocytes (ProC). (b) Visualization of non-senescent and senescent chondrocytes in the control and PM treatment groups. (c, d) SID scores in control vs. PM-treated samples (single-cell level: c; pseudobulk level: d). (e, f) SID score in SLPI^-^ vs. SLPI^+^ chondrocytes (single-cell level: e; pseudobulk level: f). (g) Boxplot of SLPI expression in non-senescent and senescent chondrocytes. (h) Trajectory of chondrocyte senescence constructed based on SID score. (i) Annotations of cells corresponding to senescence trajectories. (j) Heatmaps of genes with similar kinetic trends to senescence trajectories. Data are presented as mean ± standard deviation. *p < 0.05; **p < 0.01; ***p < 0.001; ****p < 0.0001; ns, not significant. (Linear mixed-effects model: c, e).

Senescence of chondrocytes plays an important role in OA, and we can gain new insights by using seneR to analyze bulk and single-cell RNA-seq data sets of chondrocytes [20]. Chondrocytes were annotated according to the cell subset marker provided in the original text [20] (Fig. 3a). It is worth noting that, as shown in Fig. 3b, phentolamine (PM)-treated group, an a-AR antagonist available clinically, had a smaller proportion of senescent cells than the control group. To statistically account for donor-dependent effects, we employed a linear mixed-effects model (SID_score ∼ group + (1 | donor)). This robust analysis revealed that PM treatment was associated with a significant reduction in SID scores at the single-cell level (β = −0.074, 95 % CI [-0.091, −0.058], p = 0.015; mixed-effects model, Fig. 3c). This finding was corroborated by a donor-level pseudobulk analysis, which showed a consistent trend (Fig. 3d).

Given the reported role of α2-AR signaling in promoting hypertrophic degeneration via secretory leukocyte protease inhibitor (SLPI) [20], we hypothesized that PM may alleviate senescence, with SLPI potentially acting as a downstream correlate. Supporting this, SLPI^+^ cells exhibited significantly higher SID scores than SLPI^-^ cells (β = 0.061, 95 % CI [0.047, 0.073], p < 2e-16; mixed-effects model) (Fig. 3e, f). Consistently, SLPI expression was significantly elevated in senescent versus non-senescent cells (Fig. 3g). A concordant, though non-significant, trend was observed in independent bulk RNA-seq data (Figure S2a-c). As an α2-AR antagonist, PM treatment significantly downregulated ADRA2A (α2-AR canonical marker, p_adj=0.0418) and concurrently reduced SLPI expression (p_adj<0.0001) (Figure S2d). Notably, ADRA2A and SLPI expression showed a strong positive correlation across all samples (R²=0.9207, p = 0.0024; Figure S2e), indicating their co-regulation within the α2-AR signaling pathway. Collectively, these results position PM as a promising therapeutic candidate for delaying chondrocyte senescence.

In addition, seneR can analyze the trajectory of cellular senescence. In this case study of chondrocytes, we found that proliferation chondrocytes (ProC) are at the root of the trajectory and hypertrophic chondrocytes (HTC) and fibrocartilage chondrocytes (FibC) are at the end (Fig. 3h-i), consistent with what is known about cartilage development [21], [22], [23]. To ensure the robustness and independence of our senescence trajectory, we performed validation using Slingshot trajectory inference on a gene set that excluded all genes used in SID computation (Figure S2f-g). This data-driven approach identified three highly concordant trajectories (pairwise Spearman's ρ ≈ 1), all of which showed statistically significant positive correlations with our SID-based pseudotime (Trajectory 1: ρ = 0.29, p < 2.2e-16; Trajectory 2: ρ = 0.36, p < 2.2e-16; Trajectory 3: ρ = 0.31, p < 2.2e-16; Figure S2h). The convergence of evidence from multiple independent trajectories provides strong validation that the chondrocyte senescence progression we identified represents a genuine biological pattern, rather than being dependent on the specific gene set used in SID computation. The moderate correlation strengths (ρ = 0.29–0.36) reflect the expected biological complexity, where senescence represents one major, but not exclusive, driver of transcriptomic changes in osteoarthritic chondrocytes.

We analyzed genes that follow similar kinetic trends with senescence trajectories. As shown in Fig. 3j, CDKN1C (Cyclin Dependent Kinase Inhibitor 1 C), a negative regulator of cell proliferation, increases along the aging trajectory, while HMGB2 (High Mobility Group Box 2) decreases. This pattern is consistent with previous studies, which have identified the upregulation of CDKN1C as a hallmark of senescence, leading to cell cycle arrest [24], [25]. Meanwhile, the decline in HMGB2 results in the stiffening of chromatin structure through the formation of senescence-associated heterochromatin foci (SAHF), thereby accelerating cellular senescence [26], [27]. For chondrocytes, the changes of COL10A1, COL11A1, COL9A2, and COL9A1 indicate that the ECM of chondrocytes also changes during senescence, and the hardening or abnormal remodeling of ECM may indirectly promote senescence [28], [29], [30].

### 2.4 Verification of the therapeutic effect of PM in delaying the senescence of chondrocytes

**Fig. 4:**
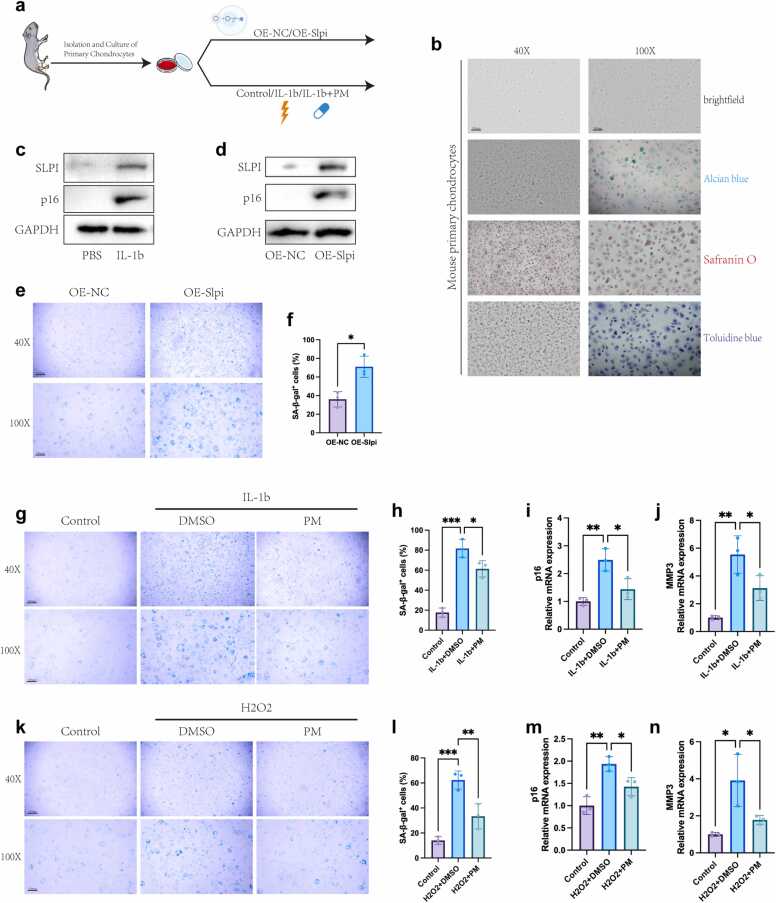
Verification of the therapeutic effect of PM in delaying the senescence of chondrocytes. (a) Schematic diagram of primary chondrocyte isolation and in vitro experimental design. (b) Representative images of brightfield, Alcian blue, Safranin O, and Toluidine blue staining of chondrocytes. (c, d) Protein levels of p16 and SLPI detected by Western blot (WB) after IL-1β treatment (c) or Slpi overexpression (OE-Slpi; d). (e, f) Representative images of SA-β-gal staining (e) and quantitative analysis of positive cells (f) in OE-negative control (OE-NC) or OE-Slpi chondrocytes. (g, h) Representative images of SA-β-gal staining (g) and quantitative analysis of positive cells (h) in chondrocytes treated with 10 ng/ml IL-1β alone or combined with 1 μM PM. (i, j) Quantitative real-time PCR (qPCR) analysis of p16 and MMP3 mRNA expression in chondrocytes treated with 10 ng/ml IL-1β alone or combined with 1 μM PM. (k, l) Representative images of SA-β-gal staining (k) and quantitative analysis of positive cells (l) in chondrocytes treated with H₂O₂ alone or combined with 1 μM PM. (m, n) qPCR analysis of p16 and MMP3 mRNA expression in chondrocytes treated with H₂O₂ alone or combined with 1 μM PM. Data are presented as mean ± standard deviation (n = 3 independent experiments). *p < 0.05; **p < 0.01; ***p < 0.001; ****p < 0.0001; ns, not significant. (Student’s *t*-test: f; ANOVA: h, i, j, l, m, n). OE, overexpression; NC, negative control.

To verify the therapeutic effect of PM in delaying chondrocyte senescence, we extracted primary articular chondrocytes from mice for experimental validation (Fig. 4a). Observation and staining of the glycosaminoglycans in the extracellular matrix (ECM) ensure that chondrocytes are in a suitable condition (Fig. 4b). After stimulation with IL-1β, we detected elevated levels of both p16 and SLPI via WB (p16: 9.03 ± 2.10-fold vs. control; SLPI: 2.70 ± 0.66-fold vs. control; n = 3, p < 0.001), suggesting that SLPI could serve as a potential marker of chondrocyte senescence (Fig. 4c). To further investigate the role of SLPI, we constructed an SLPI overexpression plasmid and transfected it into chondrocytes. Both WB and SA-β-gal staining results indicated that SLPI overexpression led to increased chondrocyte senescence (p16: 31.96 ± 22.2-fold vs. OE-NC; SLPI: 4.67 ± 1.72-fold vs. OE-NC; n = 3, p < 0.001; Fig. 4d-f).

To systematically evaluate the therapeutic potential of PM, we first investigated its dose-response and time-course effects on IL-1β-induced chondrocyte senescence. Dose-response analysis revealed that PM alleviated senescence in a concentration-dependent manner, with significant effects observed from 0.5 μM and a maximal effect at 1–5 μM (Figure S2j). Time-course experiments demonstrated that the anti-senescence effect of 1 μM PM became statistically significant after 24 h of treatment and was sustained at 48 h (Figure S2k). Critically, cell viability assays confirmed that PM at these effective concentrations (0.1–5 μM) did not induce significant cytotoxicity over 24 h (Figure S2i), indicating that its beneficial effect is not attributable to reduced cell survival. Based on these results, we selected 1 μM PM for 24 h as a robust and non-toxic treatment for subsequent validation. Consistent with our hypothesis, IL-1β and H2O2 stimulation significantly increased markers of senescence (including the number of SA-β-gal-positive cells, transcriptional level of p16, and SASP factor MMP3 expression), respectively, and PM treatment effectively reversed this trend (Fig. 4g-n). These findings collectively demonstrate that PM has the potential to delay chondrocyte senescence.

## 3 Discussion

Cellular senescence is a critical biological process implicated in a wide range of disease-related pathologies. Yet, its accurate detection remains challenging due to biomarker heterogeneity and technical limitations of conventional assays. In this study, we present seneR, an R-based computational tool that enables robust senescence assessment from transcriptomic data by integrating the validated SenCID model with enhanced functionalities for phenotype analysis and visualization. Our case studies demonstrate the utility of seneR in uncovering senescence-driven mechanisms in T2D and OA, revealing novel therapeutic insights.

seneR addresses a critical gap in senescence research by bridging Python-based models (SenCID) with R-dominated bioinformatics ecosystems (e.g., Seurat, SingleCellExperiment). This adaptation holds significant importance for several reasons. Firstly, many researchers with limited programming experience often struggle to utilize Python-based predictive models effectively. While some researchers with Python skills may attempt to integrate the results of the original model with the extensive suite of R packages for downstream analysis, this process is often problematic. For example, differences in package dependencies between Python and R can lead to inconsistencies in sample naming, complicating integrative analyses—especially when dealing with large datasets such as single-cell RNA sequencing (scRNA-seq) with thousands of samples. Additionally, the requirement for different input and output formats between the two platforms can further hinder usability, particularly when handling large volumes of data. The process of reading, converting formats, and exporting data becomes cumbersome and time-consuming. Moreover, the original Python model's strict dependency requirements restrict its use to Linux or UNIX systems, effectively excluding the majority of users who operate on Mac or Windows platforms. This limitation significantly reduces the accessibility of the model for researchers who focus solely on the biological significance of senescence.

By incorporating the predictive model into the seneR package, we addressed these limitations. The seneR package leverages the extensive R ecosystem of and ensures compatibility across multiple operating systems, including Mac and Windows. This adaptation not only enhances accessibility but also streamlines the workflow for researchers, allowing them to seamlessly integrate senescence assessments with other bioinformatics analyses within the R environment.

Our analysis of human islets revealed that age correlates with increased senescence burden. The elevated SID scores observed in islets from elderly donors reinforce the established link between aging and islet cell dysfunction, corroborating previous findings implicating cellular senescence in β-cell impairment. Notably, beyond β-cells, our study identified significantly higher SID scores in α-cells from individuals with T1D compared to controls. This observation aligns with prior reports in mouse models, which detected senescence markers (including Cdkn1a, Cdkn2a, and nuclear HMGB1 loss) in α-cells of T1D mice [31]. While diabetes research has traditionally focused on β-cells, α-cell dysfunction is critically important, particularly in T1D, where dysregulated glucagon secretion is a prominent feature [32]. Our findings suggest that senescence within α-cells may contribute to this glucagon secretion abnormality in T1D, although this hypothesis warrants further experimental validation.

Limitations of conventional senescence detection methods have precluded detailed investigation into the impact of cellular senescence on dynamic insulin secretion patterns, especially in response to stimulation by distinct macronutrient classes (carbohydrates, amino acids, and fatty acids). Leveraging our newly developed tools and multi-omics data, this study directly addresses this gap. Our results demonstrate that islet cells with higher senescence levels generally exhibit a diminished capacity for dynamic insulin secretion across these nutrient stimuli compared to their less senescent counterparts. This diminished responsiveness is consistent with previous reports of age-associated declines in β-cell glucose sensitivity [33], [34]. While further experimental confirmation is needed, these findings collectively highlight the potential of our methodological approach to be applied in multiple fields.

In a case study of OA, seneR identified that SLPI expression and PM treatment are both closely associated with SID scores. Complementary to these computational findings, our in vitro experiments further validated the functional relevance of SLPI and PM in regulating chondrocyte senescence. These results are consistent with prior [20]. Trajectory analysis further reveals chondrocyte subsets and gene expression that change with senescence levels. Trajectory analysis further delineated the progression of chondrocyte senescence, revealing a differentiation continuum from progenitor chondrocytes (ProC) to terminally differentiated hypertrophic (HTC) and fibroblastic (FibC) states—cell populations previously associated with advanced senescence and OA [21], [22], [23]. The identification of senescence-related genes has found potential chondrocyte senescence treatment targets such as CDKN1C and HMGB2. Further, in vitro validation functionally confirmed seneR's computational predictions: SLPI significantly increased under IL-1β stimulation, its overexpression also led to chondrocyte senescence, and PM treatment could reverse IL-1β-induced chondrocyte senescence. Given PM's established clinical safety profile as an α-blocker, these findings accelerate its translational repurposing potential for OA therapy.

In summary, our study introduces seneR as a powerful and accessible tool for assessing cellular senescence from transcriptomic data, bridging the gap between Python-based models and the R-dominated bioinformatics community. Through comprehensive case studies in T2D and OA, we have demonstrated the utility of seneR in uncovering novel senescence-related mechanisms and identifying potential therapeutic targets. Our findings highlight the importance of cellular senescence in age-related diseases and underscore the potential of seneR to facilitate further research in this field. Future work will focus on optimizing the seneR model and exploring its applications in a broader range of biological contexts to enhance our understanding of cellular senescence and its therapeutic implications.

## 4 Methods

### 4.1 Assessment of senescence and senescence-related phenotypes

seneR is written and packaged in R programming language (version 4.4.2), and its source code and tutorials can be viewed on github (https://github.com/dr-yi-zhang/seneR). The core senescence prediction model within seneR is the pre-trained SenCID model as published by Tao et al. [14]. The model weights and architecture are used verbatim from the authors' original public repository (https://github.com/JackieHanLab/SenCID), which is distributed under an MIT License. This model was trained using machine learning on a dataset comprising 602 samples from 52 senescence-related studies, with details available in the original [14]. Originally implemented in Python, the model classifies input transcriptomic count matrices into six distinct senescence modes. It then assesses the degree of senescence in each sample based on senescence-related genes associated with these modes.

Inputs for both bulk and single-cell transcriptome analysis are counts matrix. Based on the evaluated SID score, the monocle R package is used to plot the trajectory of senescent cells, and the genes that change along the trajectory are visualized.

For the assessment of senescence-related phenotypes, we collected aging-associated genes summarized on the Aging Atlas website (https://ngdc.cncb.ac.cn/aging) [35]. These genes were categorized into several gene sets according to their corresponding phenotypes. The enrichment levels of these phenotypes were evaluated using the Gene Set Variation Analysis (GSVA) method.

To facilitate accessibility for users without programming expertise, we developed an interactive web application using the Shiny framework (version 1.8.0). The application is hosted at https://lzy-rproject.shinyapps.io/seneR-analysis-portal/ and provides a point-and-click interface for all core seneR functionalities, including SID score calculation, phenotype assessment, and result visualization. The backend of the application directly calls the seneR R package functions to ensure analytical consistency with the command-line interface.

### 4.2 Benchmarking against SenCID

To validate the equivalence of seneR with the original SenCID model, we performed comprehensive benchmarking on the test datasets from the original publication (GSE94980, https://github.com/JackieHanLab/SenCID/blob/main/SenCID/demo/demo/origin_matrix_GSE94980.txt). We compared the output results of seneR and the original SenCID by analyzing the correlation of the per-sample SID score, the receiver operating characteristic curve (ROC), calibration curves, and confusion matrices.

### 4.3 Senescence Identity (SID) Score

The SID score is a normalized probability metric (range: 0–1) quantifying cellular senescence levels, derived from two core computational steps based on transcriptomic data (detailed algorithmic details refer to Tao et al. [14]): First, a Support Vector Machine (SVM) integrated with Recursive Feature Elimination (RFE) is applied to screen cell type-specific senescence-associated feature genes, generating a raw decision value that reflects the similarity of the sample to senescent phenotypes. Second, a logistic regression transformation is used to normalize the raw decision value to the [0,1] range (formula: SID score=$\frac{1}{1+e^{-\left(\alpha \cdot f\left(x\right)+\beta \right)}}$), where f(x) is the raw SVM decision value, and α and β are calibration parameters.

The SID score ranges strictly from 0 to 1 (0 = minimal senescent features; 1 = maximal senescent features) and uses only one monotonic transformation (logistic regression) to maintain the relative ranking of senescence levels. The SID score is comparable across datasets, guaranteed by: (1) unified preprocessing of input data (log2(CPM+1) transformation + z-score normalization); (2) consistent parameter calibration using a shared training cohort; (3) validated robustness across independent datasets. For detailed algorithmic implementations (e.g., feature gene screening criteria, parameter calibration process), see the original SenCID study [14].

Senescent and non-senescent cells were classified using a fixed SID score threshold of 0.5 (SID score > 0.5 = senescent cells; SID score ≤ 0.5 = non-senescent cells). This threshold was calibrated using the original SenCID training cohort (602 samples from 30 cell types) and accuracy was evaluated by cross-validation. External validation across independent datasets (including lung fibroblasts, skin fibroblasts, umbilical vein endothelial cells, and chondrocytes) confirmed consistency with the experimental gold standard (SA-β gallon staining, growth curve analysis).

### 4.4 Data collection for case studies

For the T2D case study, the dataset was obtained from the original publications [36] and the HumanIslets website (https://www.humanislets.com/). For the OA case study, the datasets were sourced from the original publications and the NIH Gene Expression Omnibus (GEO) (GSE249229, GSE271815). Additionally, the dataset GSE246425, which was used as an example, was also downloaded from the GEO database.

### 4.5 Trajectory validation analysis

Trajectory independence was validated using Slingshot (version 2.7.0) on a held-out gene set that excluded all genes used in SID score computation. We performed trajectory inference using default parameters and evaluated concordance between Slingshot pseudotime and SID-based pseudotime using Spearman's rank correlation. Internal consistency among Slingshot trajectories was assessed using pairwise correlation analysis.

### 4.6 Cell experiments

Mouse primary chondrocytes were isolated from the knee joints of 5–7 days old male C57BL/6 mice. After sacrificing the mice, the surface of the body was disinfected with 75 % ethanol. The knee joint capsule was then carefully opened, and the articular cartilage was excised and transferred into DMEM medium (dulbecco's modified eagle medium, Gibco) containing 0.2 % NB4 collagenase (absin, China) for overnight digestion. The next day, the digested tissue was filtered to remove any residual debris, and the isolated cells were cultured in DMEM supplemented with 10 % fetal bovine serum (FBS, Gibco).

For over-expression SLPI, chondrocytes were transfected with pcDNA3.1/Slpi full-length plasmid using Lipo8000 (Beyotime) according to the manufacturer’s instructions.For IL-1β treatment, mouse primary chondrocytes were treated with recombinant mouse IL-1β (10 ng/ml, MedChemExpress) for 24 h. For H₂O₂-induced senescence, mouse primary chondrocytes were treated with H₂O₂ (200 μM, MKBio Shanghai) for 2 h. For PM treatment, mouse primary chondrocytes were treated with or without PM (MedChemExpress), dissolved in DMSO (Dimethyl sulfoxide, MedChemExpress). For dose-response experiments, IL-1β-stimulated chondrocytes were treated with a range of PM concentrations (0.1, 0.5, 1, and 5 µM) for 24 h. For time-course experiments, IL-1β-stimulated chondrocytes were treated with 1 µM PM for 12, 24, and 48 h. In all PM treatment experiments, an equivalent volume of DMSO was used as the vehicle control. To assess the potential cytotoxicity of PM, cell viability was evaluated using the Cell Counting Kit-8 (CCK-8, Beyotime, China) on chondrocytes treated with the same range of PM concentrations (0.1–5 µM) for 24 h, according to the manufacturer's instructions.

### 4.7 Western blot (WB)

Protein samples were extracted using RIPA lysis buffer (MedChemExpress) supplemented protease inhibitors (MedChemExpress). Proteins were separated by electrophoresis on a 10 % SDS-PAGE gel and transferred onto polyvinylidene fluoride (PVDF) membranes (Millipore). The membranes were blocked with 5 % nonfat milk (Epizyme Biotech) and then incubated with primary antibodies at 4 °C overnight. The following primary antibodies were used: anti-p16 (MedChemExpress, HY-P86438), anti-SLPI (proteintech, 32205–1-AP), and anti-GAPDH (proteintech, 60004–1-Ig). After being washed, the membranes were incubated with horseradish peroxidase (HRP)-conjugated secondary antibodies (proteintech, SA00001–2) for 1 h at room temperature. The protein bands were visualized using enhanced chemiluminescence reagents (Thermo Fisher). All WB experiments were repeated independently for 3 times, and protein band intensities were quantified using ImageJ software (version 1.53j) with GAPDH as the internal reference. Data are presented as mean ± SD of 3 independent experiments.

### 4.8 Senescence-associated β-galactosidase (SA-β-gal) assays

Chondrocytes were fixed for 15 min and subsequently incubated with the staining reagents from the SA-β-galactosidase (SA-β-gal) assay kit (Beyotime) for an overnight period at 37°C. All SA-β-gal staining experiments were performed in 3 independent biological replicates. Cellular images were captured systematically from five pre-defined fields per well using an optical microscope. The number of SA-β-gal-positive cells was quantified in a blinded manner by two independent researchers using ImageJ software (version 1.53j). Data are presented as mean ± SEM from three independent experiments.

### 4.9 Statistical analysis

Statistical analysis was performed using GraphPad Prism 9.0 software or (GraphPad Software, USA) and R (4.4.2). The results are presented as the mean ± standard deviation (SD). Differences between groups were analyzed using Student’s *t*-test or one-way analysis of variance (ANOVA). For the human islet data analysis, linear mixed-effects models were fitted using the lmer function from the lmerTest package in R. The model included batch as a random effect, with age, sex, BMI, and T2D status as fixed effects. P-values for fixed effects were adjusted for multiple testing using the Benjamini-Hochberg FDR procedure. A p-value < 0.05 was considered statistically significant.

## CRediT authorship contribution statement

**Yunfeng Yang:** Writing – review & editing, Supervision. **Xin Ma:** Writing – review & editing. **Yao Lu:** Writing – review & editing. **Bing Li:** Writing – review & editing, Supervision, Investigation. **Cheng Chen:** Data curation. **Xinming Zhang:** Validation. **Yi Zhang:** Writing – original draft.

## Consent for publication

Written informed consent was obtained from all participants.

## Ethics approval and consent to participate

The study was reviewed and approved by the Institutional Review Board of Shanghai Tongji Hospital (K-KYSB-2021–001) and conducted in accordance with the Helsinki Declaration.

## Funding

The study is sponsored by Shanghai Committee of Science and Technology (Grant No. 22S31900300) and Shanghai Committee of Science and Technology (Grant No. 21ZR1458500).

## Declaration of Competing Interest

The authors declare that the research was conducted in the absence of any commercial or financial relationships that could be construed as a potential conflict of interest.

## Data Availability

Data generated or analyzed during this study are included in this published article and its supplementary files.

## Appendix A Supplementary material

Supplementary material

**Supplementary** Figure 1 Benchmarking of seneR and analysis of senescence-associated phenotypes. (a-d) Head-to-head benchmarking of seneR against the original SenCID using SenCID’s demo dataset (GSE94980), with SenCID’s output as the gold standard. (a) Scatter plot with linear fitting of SID5 scores from seneR and SenCID. (b) Confusion matrix for senescent/non-senescent cell classification. (c) ROC curve for senescent/non-senescent cell classification. (d) Calibration curve. (e-g) Benchmarking in the chondrocyte senescence dataset (GSE246425). (e) Correlation heatmap between seneR-derived SID scores and established senescence marker genes (expression values as TPM; black numbers in the upper triangle indicate p-values). (f, g) ROC curve (f) and confusion matrix (g) for classifying young (passage 1–3) vs. old (long-term passaged) chondrocytes using seneR’s binarization function. (h) Comparison of senescence-associated phenotypes between non-type 2 diabetes (non-T2D) and T2D islets. (i) Quantitative analysis of insulin secretion parameters: time to peak insulin and peak insulin value. Data are presented as mean ± standard deviation. *p < 0.05; **p < 0.01; ***p < 0.001; ****p < 0.0001; ns, not significant (Pearson correlation analysis: a, e; Student’s *t*-test: i; *t*-test with FDR correction: h)

**Supplementary Figure 2** (a-c) Box plots of SID scores in chondrocytes: control vs. SLPI knockout (a); COL10 negative vs. COL10 positive groups (b, COL10 is an important biomarker of chondrocyte hypertrophy); control vs. PM treatment (c). (d) Heatmap of SLPI and ADRA2A expression (TPM values) in control vs. PM-treated chondrocytes. (e) Correlation between SLPI and ADRA2A expression. (f-g) Slingshot trajectory inference based on a gene set excluding all genes used in SID computation. (h) Correlation heatmap between Slingshot trajectory pseudotime and SID-based pseudotime. (i) Quantitative analysis of CCK-8 assay in chondrocytes treated with different concentrations of PM for 24 h. (j) Quantitative analysis of SA-β-gal-positive cells in chondrocytes treated with different concentrations of PM for 24 h. (k) Quantitative analysis of SA-β-gal-positive cells in chondrocytes treated with 1 μM PM for different durations. Data are presented as mean ± standard deviation. *p < 0.05; **p < 0.01; ***p < 0.001; ****p < 0.0001; ns, not significant (Pearson correlation analysis: e; Student’s *t*-test: a-c; ANOVA: i-k)
