# Erratum to “seneR: An R package for comprehensive senescence assessment and its application in type 2 diabetes and osteoarthritis”

**DOI:** 10.34133/csbj.0210

**Published:** 2026-09-30

**Authors:** Yi Zhang, Xinming Zhang, Cheng Chen, Bing Li, Yao Lu, Xin Ma, Yunfeng Yang

**Affiliations:** ^1^ Department of Orthopedics, Tongji Hospital, School of Medicine, Tongji University, Shanghai 200092, China.; ^2^ Department of Chemistry, Rice University, Houston, TX 77005, USA.; ^3^ Department of Orthopaedics, Shanghai Sixth People’s Hospital Affiliated to Shanghai Jiao Tong University, School of Medicine, 600 Yishan Rd, Shanghai 200233, China.; ^4^ Department of Orthopedics, Ruijin Hospital, Shanghai Jiao Tong University School of Medicine, Shanghai, China.; ^5^ Department of Microbiology and Immunology, Mcgill University, Canada.

In the originally published version of “seneR: An R package for comprehensive senescence assessment and its application in type 2 diabetes and osteoarthritis,” Fig. 4g contained an inadvertent figure-assembly artifact in the IL-1β + DMSO SA-β-gal staining images at 40× and 100× magnification. During final figure preparation, duplicate copies of the same image layers were unintentionally stacked with a slight horizontal offset, resulting in a narrow duplicated/overlapped strip along the right edge of these panels [1].

**Fig. 4.**
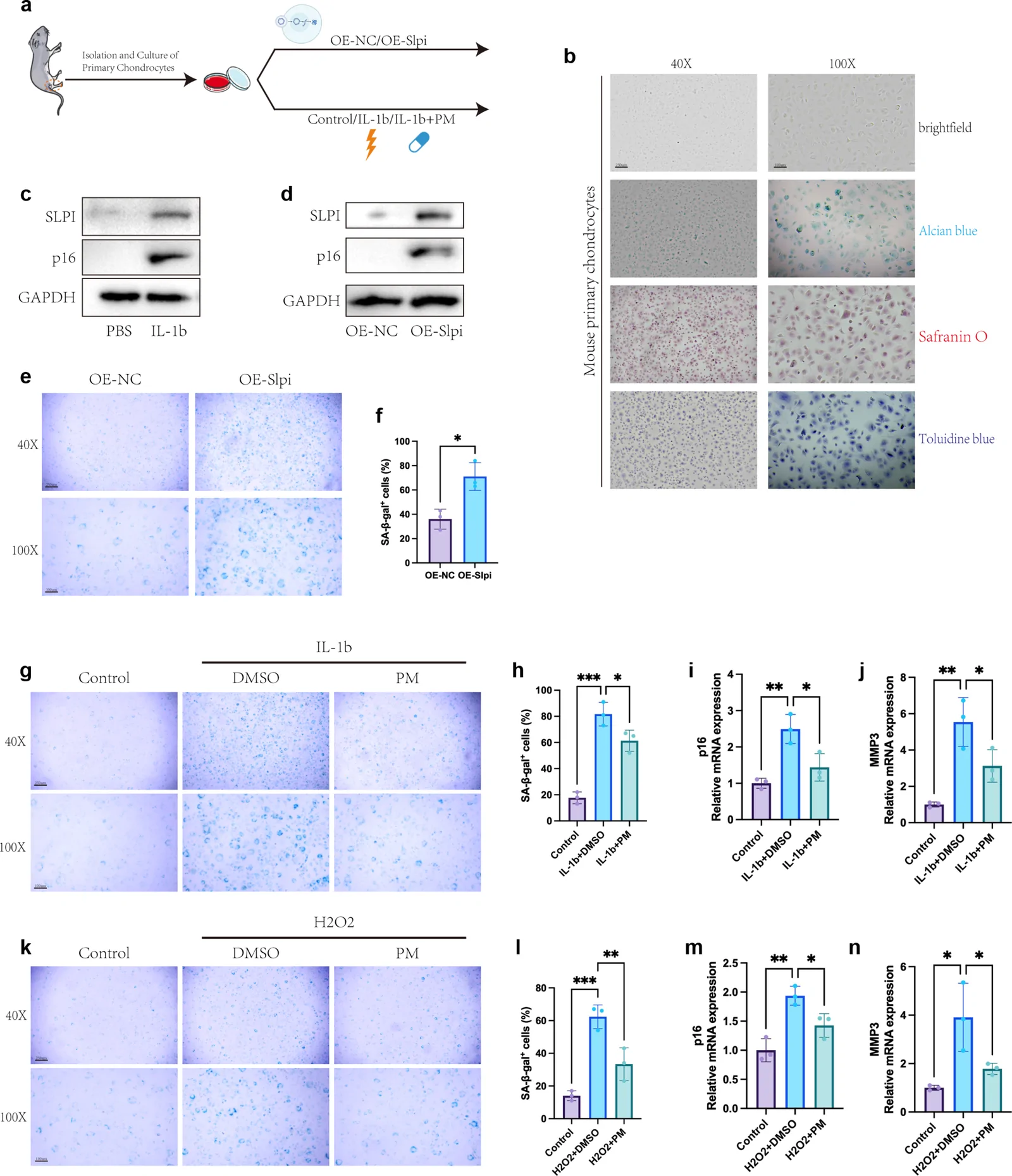
Verification of the therapeutic effect of PM in delaying the senescence of chondrocytes. (a) Schematic diagram of primary chondrocyte isolation and in vitro experimental design. (b) Representative images of brightfield, Alcian blue, Safranin O, and Toluidine blue staining of chondrocytes. (c, d) Protein levels of p16 and SLPI detected by Western blot (WB) after IL-1β treatment (c) or Slpi overexpression (OE-Slpi; d). (e, f) Representative images of SA-β-gal staining (e) and quantitative analysis of positive cells (f) in OE-negative control (OE-NC) or OE-Slpi chondrocytes. (g, h) Representative images of SA-β-gal staining (g) and quantitative analysis of positive cells (h) in chondrocytes treated with 10 ng/ml IL-1β alone or combined with 1 μM PM. (i, j) Quantitative real-time PCR (qPCR) analysis of p16 and MMP3 mRNA expression in chondrocytes treated with 10 ng/ml IL-1β alone or combined with 1 μM PM. (k, l) Representative images of SA-β-gal staining (k) and quantitative analysis of positive cells (l) in chondrocytes treated with H_2_O_2_ alone or combined with 1 μM PM. (m, n) qPCR analysis of p16 and MMP3 mRNA expression in chondrocytes treated with H_2_O_2_ alone or combined with 1 μM PM. Data are presented as mean ± standard deviation (n = 3 independent experiments). *p < 0.05; **p < 0.01; ***p < 0.001; ****p < 0.0001; ns, not significant. (Student’s *t*-test: f; ANOVA: h, i, j, l, m, n). OE, overexpression; NC, negative control.

Fig. 4 has been corrected below and in the original HTML and PDF.

This correction is limited to figure presentation and does not affect the quantified data, experimental results, interpretation, or scientific conclusions of the article. The authors sincerely apologize for this oversight and for any inconvenience it may cause to readers.
