# Supplementary figures and images for: seneR: An R package for comprehensive senescence assessment and its application in type 2 diabetes and osteoarthritis
Source: Comput Struct Biotechnol J. 2025 Dec 31;31:192–201. doi: 10.1016/j.csbj.2025.12.031 (PMC12809408; doi:10.1016/j.csbj.2025.12.031)

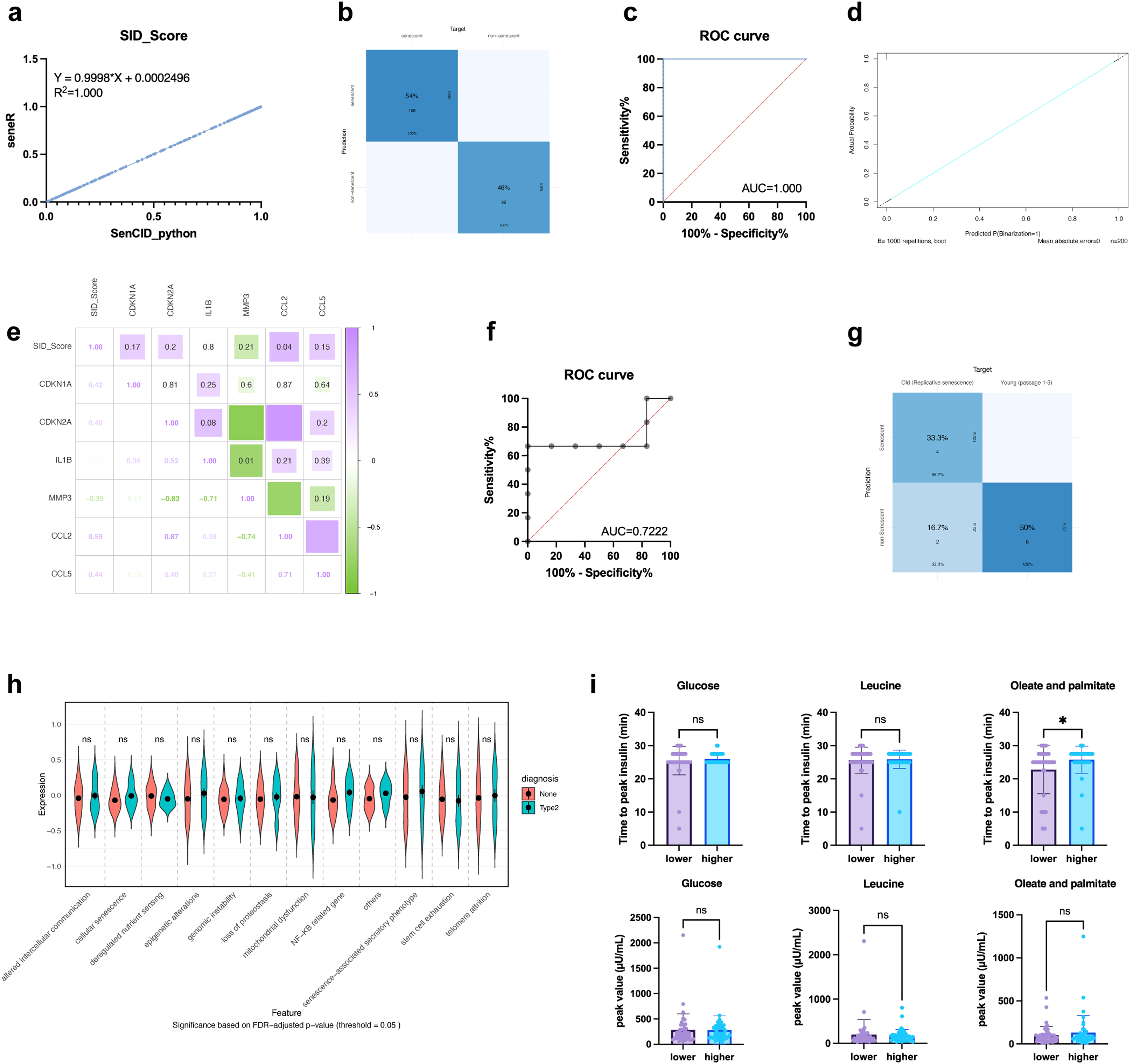

Supplement: Supplementary file 2 — Supplementary Figure 1 Benchmarking of seneR and analysis of senescence-associated phenotypes. (a-d) Head-to-head benchmarking of seneR against the original SenCID using SenCID’s demo dataset (GSE94980), with SenCID’s output as the gold standard. (a) Scatter plot with linear fitting of SID5 scores from seneR and SenCID. (b) Confusion matrix for senescent/non-senescent cell classification. (c) ROC curve for senescent/non-senescent cell classification. (d) Calibration curve. (e-g) Benchmarking in the chondrocyte senescence dataset (GSE246425). (e) Correlation heatmap between seneR-derived SID scores and established senescence marker genes (expression values as TPM; black numbers in the upper triangle indicate p-values). (f, g) ROC curve (f) and confusion matrix (g) for classifying young (passage 1–3) vs. old (long-term passaged) chondrocytes using seneR’s binarization function. (h) Comparison of senescence-associated phenotypes between non-type 2 diabetes (non-T2D) and T2D islets. (i) Quantitative analysis of insulin secretion parameters: time to peak insulin and peak insulin value. Data are presented as mean ± standard deviation. *p < 0.05; **p < 0.01; ***p < 0.001; ****p < 0.0001; ns, not significant (Pearson correlation analysis: a, e; Student’s t-test: i; t-test with FDR correction: h) [file mmc2.jpg]

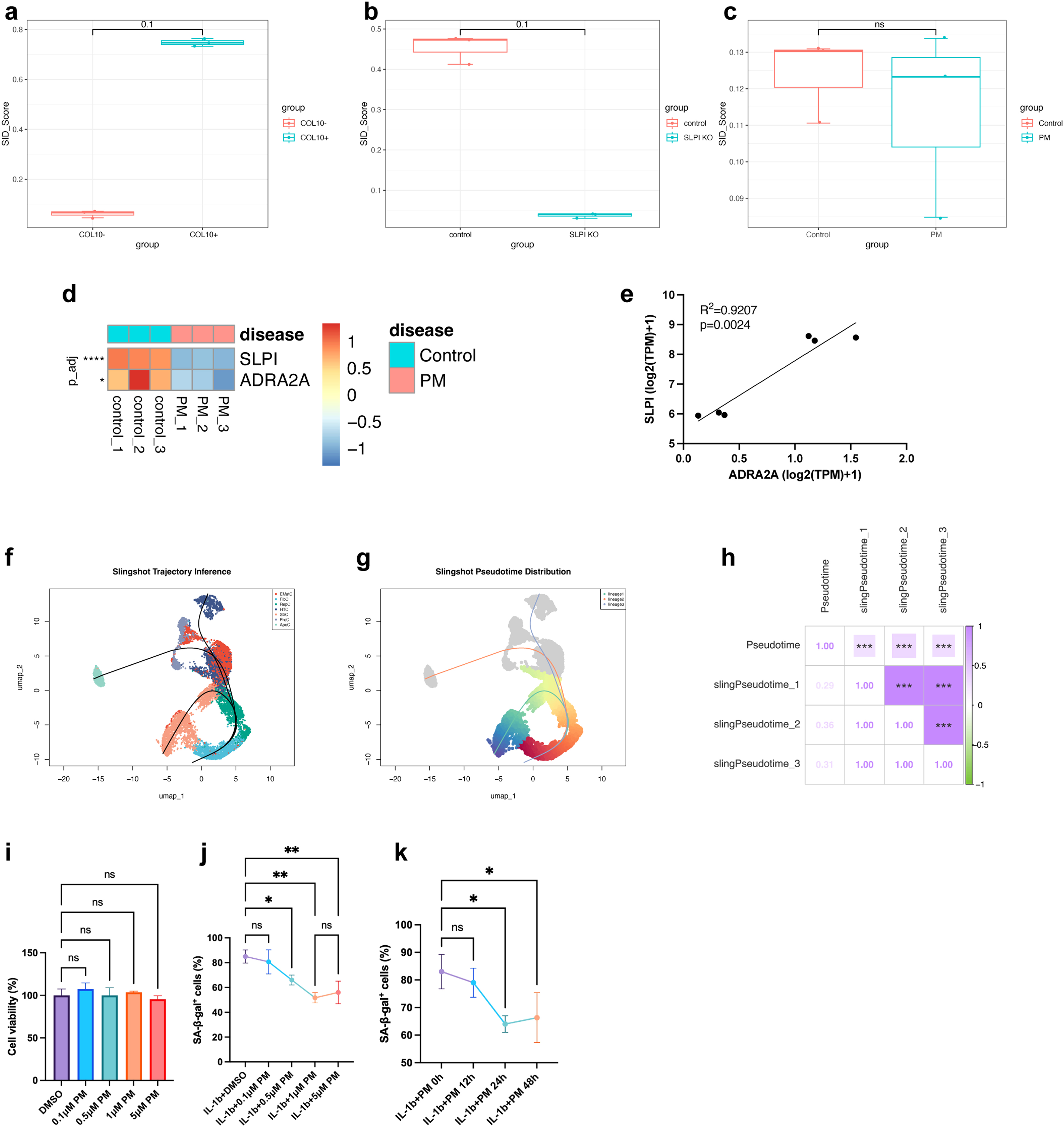

Supplement: Supplementary file 3 — Supplementary Figure 2 (a-c) Box plots of SID scores in chondrocytes: control vs. SLPI knockout (a); COL10 negative vs. COL10 positive groups (b, COL10 is an important biomarker of chondrocyte hypertrophy); control vs. PM treatment (c). (d) Heatmap of SLPI and ADRA2A expression (TPM values) in control vs. PM-treated chondrocytes. (e) Correlation between SLPI and ADRA2A expression. (f-g) Slingshot trajectory inference based on a gene set excluding all genes used in SID computation. (h) Correlation heatmap between Slingshot trajectory pseudotime and SID-based pseudotime. (i) Quantitative analysis of CCK-8 assay in chondrocytes treated with different concentrations of PM for 24 h. (j) Quantitative analysis of SA-β-gal-positive cells in chondrocytes treated with different concentrations of PM for 24 h. (k) Quantitative analysis of SA-β-gal-positive cells in chondrocytes treated with 1 μM PM for different durations. Data are presented as mean ± standard deviation. *p < 0.05; **p < 0.01; ***p < 0.001; ****p < 0.0001; ns, not significant (Pearson correlation analysis: e; Student’s t-test: a-c; ANOVA: i-k) [file mmc3.jpg]
